# Synonymous Codon Usage Bias in Plant Mitochondrial Genes Is Associated with Intron Number and Mirrors Species Evolution

**DOI:** 10.1371/journal.pone.0131508

**Published:** 2015-06-25

**Authors:** Wenjing Xu, Tian Xing, Mingming Zhao, Xunhao Yin, Guangmin Xia, Mengcheng Wang

**Affiliations:** The Key Laboratory of Plant Cell Engineering and Germplasm Innovation, Ministry of Education, School of Life Science, Shandong University, 27 Shandanan Road, Jinan, Shandong 250100, China; Saint Mary's University, CANADA

## Abstract

Synonymous codon usage bias (SCUB) is a common event that a non-uniform usage of codons often occurs in nearly all organisms. We previously found that SCUB is correlated with both intron number and exon position in the plant nuclear genome but not in the plastid genome; SCUB in both nuclear and plastid genome can mirror the evolutionary specialization. However, how about the rules in the mitochondrial genome has not been addressed. Here, we present an analysis of SCUB in the mitochondrial genome, based on 24 plant species ranging from algae to land plants. The frequencies of NNA and NNT (A- and T-ending codons) are higher than those of NNG and NNC, with the strongest preference in bryophytes and the weakest in land plants, suggesting an association between SCUB and plant evolution. The preference for NNA and NNT is more evident in genes harboring a greater number of introns in land plants, but the bias to NNA and NNT exhibits even among exons. The pattern of SCUB in the mitochondrial genome differs in some respects to that present in both the nuclear and plastid genomes.

## Introduction

The mitochondrion is a major double-membrane organelle found in most eukaryotic cells [1]. It is the site for supplying cellular energy as well as signaling, cellular differentiation, cell death and maintaining the control of the cell cycle and cell growth [2]. In one cell, unlike nuclear genome with a signal copy, there have many mitochondria, and each mitochondrion possesses multiple copies of a circular genome [3]. The mitochondrial genome is about 15–17 kb in mammals and exhibited much greater variation in size among fungi and plants [4], much smaller than the nuclear genome. There have dozens of genes in the mitochondrial genomes, encoding rRNA, tRNA and proteins functioning in energy metabolism [3].

Eukaryotic mitochondria are believed to have originated from a symbiotic relationship with bacteria, and since their incorporation, most of their genetic content appears to have either been deleted or transferred into the nuclear genome during the whole evolution course of organisms [5]. The loss and transfer into nuclear genome of mitochondrial genes are along with DNA recombination and breakage. DNA recombination and breakage, alike insertion and deletion (indel), have proved to result in genomic shock [6]. This leads to the induction of local single nucleotide substitution [7], which produces SCs and nonsynonymous codons for protein encoding genes.

Codon degeneracy arises from the encoding of each amino acid (with the exception of Met and Trp) by two or more so-called “synonymous codons” (SCs). Besides the nuclear genome, SCUB has been identified in the organellar genome, as well. SC frequency varies not just between organisms, but even between genes within an organism [8]. Termed “synonymous codon usage bias” (SCUB) has been identified in both the nuclear and organellar genome. SCUB is thought to reflect the net outcome of mutation, genetic drift and natural selection [9–12]. The evolution of plants always follows the evolution of their genomes, and DNA duplication and recombination are the major drivers of genome evolution in plants [13]. Our previous work demonstrated that SCUB of the nuclear genome is closely associated with plant evolution [14]. Moreover, we recently found that there also has an association between SCUB of the plastid genome and plant evolution [15]. Although SCUB in plant mitochondrial genomes has been widely researched [16–20], whether it, like its equivalent nuclear and plastid genomes, is different in the mitochondiral genomes from lower to higher plants has not been well addressed.

Intron gain/loss, a key component of the evolution of genomes [13], is largely driven by recombination and indel formation (Knowles and McLysaght, 2006; Sharpton et al., 2008). The likelihood of intron gain/loss at a given site has proved to be related to both the global intron frequency and the position within the gene [21]. In the nuclear genome, SCUB appears to be correlated with intron [8]. Especially, we previously demonstrated that SCUB is correlative to intron number and shows disequilibrium among exons in plants [14]. However, our recent study showed that both the correlation of SCUB frequency to intron number and its heterogeneity among exons are not present in the plastid genome [15]. Therefore, whether this relationship in the mitochondrial genome is similar to the plastid genome or nuclear genome has yet to have been documented.

Here we present an analysis of SCUB in the mitochondrial genomes present in a set of species ranging from algae to land plants with aim to answer above issues, and to compare their difference and similarity to the nuclear and plastid genomes. We found that SCUB in the mitochondrial genomes also exhibits heterogeneity among different taxonomies of plants; it is correlated to intro number in land plants but shows evenness among exons.

## Materials and Methods

### Mitochondrial genome sequences and gene structure

The mitochondrial genome sequences of 24 species were downloaded from NCBI (http://www.ncbi.nlm.nih.gov/genomes/GenomesGroup.cgi?taxid=33090&opt=organelle). The 24 selected plant species comprised four chlorophytes, four charophytes, six bryophytes (including two anthocerotophyta species, two marchantiophyta species, two bryophyta species), one pteridophyte, one gymnosperm, four monocotyledonous species, and four dicotyledonous species (Table 1). The intron/exon structure of protein coding genes was obtained from the online CDS annotation at NCBI (http://www.ncbi.nlm.nih.gov/genomes/GenomesGroup.cgi?taxid=33090&opt=organelle). The CDS sequences as well as their GI and GeneID numbers are listed in S1 Table. Coding sequences of length a multiple of three were deemed to be canonical genes for analysis. In addition to ATG, other forms of the first three nucleotides were also deemed as atypical start codons; in addition to TAA, TAG and TGA, other forms of the last three nucleotides were also assumed to represent atypical stop codons. Codons interrupted by an intron between the first and the second nucleotide were treated as belonging to the subsequent exon, while those interrupted between the second and the third nucleotides were deemed to belong to the current exon.

**Table 1:** Intron numbers in mitochondrial genes.

Taxomony	Species	Accession	Genes with introns										
			0	1	2	3	4	5	6	7	9	Total	Intronless (%)
Chlorophyta	*Oltmannsiellopsis viridis*	NC_008256	34	2								36	94.4
	*Ostreococcus tauri*	NC_008290	43									43	100.0
	*Microspora stagnorum*	NC_022862	46							1		47	97.9
	*Pseudendoclonium akinetum*	NC_005926	69	2			1					72	95.8
Charophyta	*Entransia fimbriata*	NC_022861	33	2								35	94.3
	*Mesostigma viride*	NC_008240	38	1	2							41	92.7
	*Chaetosphaeridium globosum*	NC_004118	43	1	1			1				46	93.5
	*Chara vulgaris*	NC_005255	39	3	2	1			1			46	84.8
Bryophyte	*Phaeoceros laevis*	NC_013765	24	3	5	4	2					38	63.2
	*Megaceros aenigmaticus*	NC_012651	35	2	7	4						48	72.9
	*Treubia lacunosa*	NC_016122	56	8	3	1					1	69	81.2
	*Marchantia polymorpha*	NC_001660	63	7	4	1					1	76	82.9
	*Physcomitrella patens*	NC_007945	26	10	3	2	1					42	61.9
	*Anomodon rugelii*	NC_016121	30	10	2	3	1					46	65.2
Pteridophyte	*Huperzia squarrosa*	NC_017755	132	5	5	3	2					147	89.8
Gymnosperms	*Cycas taitungensis*	NC_010303	29	4	1	1	4					39	74.4
Monocotyledon	*Butomus umbellatus*	NC_021399	23	2		1	4					30	76.7
	*Oryza sativa*	NC_007886	44	5		1	4					54	81.5
	*Zea mays*	NC_007982	152	4		1	6					163	93.3
	*Sorghum bicolor*	NC_008360	24	3		1	4					32	75.0
Dicotyledon	*Beta vulgaris*	NC_002511	133	2	1		4					140	95.0
	*Nicotiana tabacum*	NC_006581	146	5		2	3					156	93.6
	*Arabidopsis thaliana*	NC_001284	108	4		1	4					117	92.3
	*Glycine max*	NC_020455	80	3		1	4					88	90.9

### Calculation of SCUB frequency

Instead of indicators such as relative synonymous codon usage (RSCU) and codon adaptation index (CAI), we calculated the SCUB frequency according to our previous study [14] to intuitively compare the difference among species. The calculations were based on the 59 SCs encoding 18 amino acids; the five codons not considered were the three stop codons, ATG (Met) and TGG (Trp). To avoid the effect of gene length, the frequency of a given codon of 59 SCs was normalized by dividing the number of this codon to the codon number of coding DNA sequences [22]. The codon number of CDS was calculated by the number of all codons except for the start and stop codons; atypical start codons (the first three nucleotides are not ATG) and atypical stop codons (the last three nucleotides are not TAA, TAG and TGA) that are rarely present in a few plastid genes of some species [23–25] were also excluded. The total SCUB frequency based on the third position nucleotide was normalized by as the ratio of the number of all SCs having A, T, C or G at the third position (abbreviated as NNA, NNT, NNC or NNG) to the codon number of coding DNA sequences.

The SC frequency for a given amino acid was defined as the ratio of the number of its C- and G-ending SCs (NNCs/Gs) to the number of its A- and T-ending SCs (NNAs/Ts) from all CDS sequences except for atypical start and atypical stop codons in the plastid genome. The effect of the second nucleotide or first nucleotide of the following codon on SCUB frequency based on the third nucleotide of codons was defined as the ratio of the number of a certain combination to the number of the other combination. For example, the effect of A at the second position on the SCUB frequency of G- and C-ending codons was calculated as the ratio of the number of all SCs with AG as the second-third nucleotides (NAG) to the number of all SCs with AC as the second-third nucleotides (NAC); the effect of A at the first nucleotide of the following codon on the frequency of C- and G-ending codons was calculated as the ratio of the number of all SCs with CA as the third-next first nucleotides (NC|A) to the number of all SCs with GA as the third-next first nucleotides (NG|A). The SCUB frequency of C- and G-ending codos in a given amino acid that has C- and G-ending SCs was defined as the ratio of the number of G-ending codon (e.g. GCG of alanine) to the number of C-ending codon (e.g. GCC of alanine). These indices were calculated based on the ratios between the numbers of two codon sets, so the effect of gene length was automatically normalized.

Mitochondrial transcripts undergo a type of posttranscriptional processing called RNA editing, which converts specific cytidines to uridines (C-to-U) or uridines to cytidines (U-to-C) through undefined mechanisms (reviewed in [26–28]). The numbers of codons occurring C-to-U and U-to-C were both drastically smaller than the total number of codons. The codons undergoing C-to-U or U-to-C conversion include start codons, stop codons, non-encoding sequence (intron and untranslated region) and encoding codons; the conversion in encoding codons can occur at the first, second or third nucleotide. Thus, the RNA editing leads to rare conversion to SCs or non-synonymous codons, which has negligible effect when calculating SCUB frequency. Moreover, the C-to-U and U-to-C RNA editing is not annotated in the plastid genome database of most species (http://www.ncbi.nlm.nih.gov/genomes/GenomesGroup.cgi?taxid=33090&opt=organelle). Thus, following previous studies concerning SCUB of mitochondrial genome, the RNA editing is not considered when calculating SCUB frequency in this work.

### Phylogenic tree construction and principle component analysis (PCA)

We constructed a phylogenic tree with the normalized SC frequencies of 59 SCs encoding 18 amino acids based on the unweighted pair-group average (UWPGA) method (euclidean distances) in the STATISTICA software package (V6.0, StatSoft). The normalized SC frequencies of 59 SCs were subject to perform PCA based on the varimax method in the SAS software package (V9.0, SAS Institute Inc.), and scatter plot diagrams were generated from the coefficients given by the first three PCs.

### Statistical analysis

The comparison between the SCUB frequencies of 18 amino acids and 1 was performed with one-sample *t*-test. The difference in the SCUB frequencies of NNA/T from NNC/G in the plastid genome of a given plant was calculated using the numbers of NNA/T and NNC/G with the chi square (χ^2^) test. The difference in the SCUB frequencies of NNA/T and NNC/G among genes using responding SC numbers with various introns or among exons was calculated with the chi square (χ^2^) test of the cross-table analysis. The difference in the SCUB frequencies among algae, bryophytes, pteridophytes, gymnosperms, monocotyledons and dicotyledons was calculated using the ratios of responding SCs with the Kruskal-Wallis test. The difference between the SCUB frequencies of nucleotide pairs based on the third nucleotide concerning DNA methylation was analyzed with the chi square (χ^2^) test, and the difference of the ratios of NCG/NCC of Ala, Pro, Ser, Thr from those of NXG/NXC (X is G or C) of Arg, Gly, Leu and Val was analyzed with the Mann-Whitney test. The difference in the frequencies of C and G from those of A and T in the gene body, intron and whole genome sequences was evaluated with the chi square (χ^2^) test, and the difference in the ratios of NNC/G to NNA/T from those of the ratios C and G to A and T in the gene body, intron and whole genome sequences was calculated with the chi square (χ^2^) test of the cross-table analysis.

## Results

### Gene amount and intron distribution in the mitochondrial genome

We found the amount of protein coding genes (abbreviated as genes hereafter) ranged from 30 in *B*. *umbellatus* to 156 in *N*. *tabacum* (Table 1). Most genes were intronless in all of 24 selected species. In chlorophytas, intron distribution was different among four species, and genes with intron(s) were fewer than those in charophytas and land plants. In charophytas, genes possessing one intron were found in four detected species, and genes with 2~6 introns were also present in some genomes. In bryophytes, six selected species had genes containing 1~3 introns, and *P*. *laevis*, *P*. *patens* and *A*. *rugelii* also had genes harboring four introns, while genes with nine introns were present in the marchantiophyta. The intron content varied from zero to five imong the vascular plants. Among them, there had no gene with two introns in monocytoledons, In the angiosperm entries, genes containing two introns were only present in *B*. *vulgaris*; in the latter species, there has no genes containing three introns.

### Start codons, stop codons and internal stop codons in the mitochondrial genome

Besides ATG and three stop codons, atypical start (not ATG) and stop (not TAA, TGA and TAG) codons are present in some mitochondrial genes [23–25]. We found atypical start codons distributed differently in the mitochondrial genes of various taxonomies of plants (Table 2). GTG is the commonest atypical start codon present in the plastid genomes of most algae and land plants [15]. In the plant mitochondrial genomes, GTG was only present in algae, bryophytes and pteridophyte. It was the unique atypical start codons in some detected algae (chlorophytes and charophytes); in bryophytes, it was found in marchantiophyta and bryophyta species (*T*. *lacunose*, *M*. *polymorpha*; *P*. *patens*, *A*. *rugelii*) but not in anthocerotophyta species (*P*. *laevis*, *M*. *aenigmaticus*). In land plants, ACG was a common atypical start codon present in most land plants except for three detected bryophytes. Among other C- and G-ending atypical start codons, TGC, CGC and GCG were only found in bryophyte *M*. *polymorpha*, while GGG was found in gymnosperm *C*. *taitungensis* and dicotyledonous species *A*. *thaliana*. For A- and T-ending atypical start codons, except for AAA present in bryophyte *M*. *polymorpha*, others were represented in the mitochondrial genes of angiosperms.

**Table 2:** Variation in start and stop codons in the mitochondrial genomes.

Taxomony	Species	Start codon											Stop codon								
		ATG	GTG	ACG	TGC	CGC	GCG	GGG	AAA	AGA	ATA	AAT	TAA	TAG	TGA	CAA	CGA	GGT	AAA	AAT	CAG
Chlorophyta	*O*. *viridis*	34	2										27	9							
	*O*. *tauri*	43											35	2	6						
	*M*. *stagnorum*	45	2										30	10	7						
	*P*. *akinetum*	72											40	21	11						
Charophyta	*E*. *fimbriata*	35											25	6	4						
	*M*. *viride*	41											38	2	1						
	*C*. *globosum*	46											30	9	7						
	*C*. *vulgaris*	44	2										30	8	8						
Bryophyte	*P*. *laevis*	31		7									16	10	9	1	1	1			
	*M*. *aenigmaticus*	40		8									19	14	14	1					
	*T*. *lacunosa*	68	1										31	19	19						
	*M*. *polymorpha*	68	2		1	1	1		2				34	24	18						
	*P*. *patens*	40	2										24	8	10						
	*A*. *rugelii*	42	2	2									21	11	10	4					
Pteridophyte	*H*. *squarrosa*	140	1	6									54	39	45	7	2				
Gymnosperms	*C*. *taitungensis*	31		7				1					10	11	6	4	6				2
Monocotyledon	*B*. *umbellatus*	28		2									12	5	12		1				
	*O*. *sativa*	51		2						1			23	16	12		1		1	1	
	*Z*. *mays*	159		3						1			54	56	62		1				
	*S*. *bicolor*	29		2						1			14	10	7		1				
Dicotyledon	*B*. *vulgaris*	136		1							1		58	37	43	1	1				
	*N*. *tabacum*	153		3									60	49	47						
	*A*. *thaliana*	113	1	1				1				1	53	20	44						
	*G*. *max*	83		5									30	17	40		1				

TAA, TAG and TGA were the commonest stop codons in the mitochondrial genomes of 23 species (the exception was *O*. *viridis*, in which TGA was not represented) (Table 2). In the plastid genomes, atypical stop codons were mostly present in the pteridophytes with large amounts [15]. For the mitochondrial genomes, no atypical stop codon was used by any of detected algal species, but there were present in land plants from bryophytes to angiosperms. There had only one form of C- and G-ending atypical stop codon that was present in two mitochondrial genes of gymnosperm *C*. *taitungensis*. CAA was commonly used as atypical stop codons in some species of bryophytes, pteridophyte and gymnosperm, but was rarely in angiosperms (except for *B*. *vulgaris*). CGA were the common atypical stop codon in the vascular plants but not in bryophytes (except for *P*. *laevis*). Moreover, these two atypical stop codons were more preferential in pteridophyte one gymnosperm species than in bryophytes and angiosperms. For other A- and T-ending codons, GGT was only found in *P*. *laevis*, and AAA and AAT were only identified in *O*. *sativa*.

Internal stop codons rarely exist in gene body of some mitochondrial and plastid genomes [24, 29]. Unlike nuclear genes, uridine-to-cytidine (U-to-C) is a kind of RNA processing for a few mitochondrial and plastid genes in some species [30, 31]. Because U-to-C editing often acts to eliminate internal stop codons in transcripts of essential genes, it is possible to predict the activity and relative abundance of U-to-C RNA editing in a species based on the presence and abundance of internal stop codons in otherwise intact and presumably functional genes [29]. For plastid genome, internal stop codons are present in most leptosporangiate ferns, but not in either most early diverging fern lineages or seed plants [32], suggesting that U-to-C editing originated in the common ancestor of vascular plants and hornworts, with independent losses from the lycophyte *Selaginella* and most (or all) seed plants [29]. We found internal stop codons were very common in two anthocerotophyta species of bryophytes (*P*. *laevis*, *M*. *aenigmaticus*), and they were very rarely present in marchantiophyta and bryophyta species (only one in *T*. *lacunosa*) (S2 Table). However, unlike plastid genomes, internal stop codons were not rich in pteridophyte speices, and only four were present in *H*. *squarrosa*. Moreover, among the internal stop codons, the number of either TAA or TGA with A at the third position was much higher compared with TAG.

### SCUB patterns are heterogeneous among plants at different evolutionary positions

**Fig 1:**
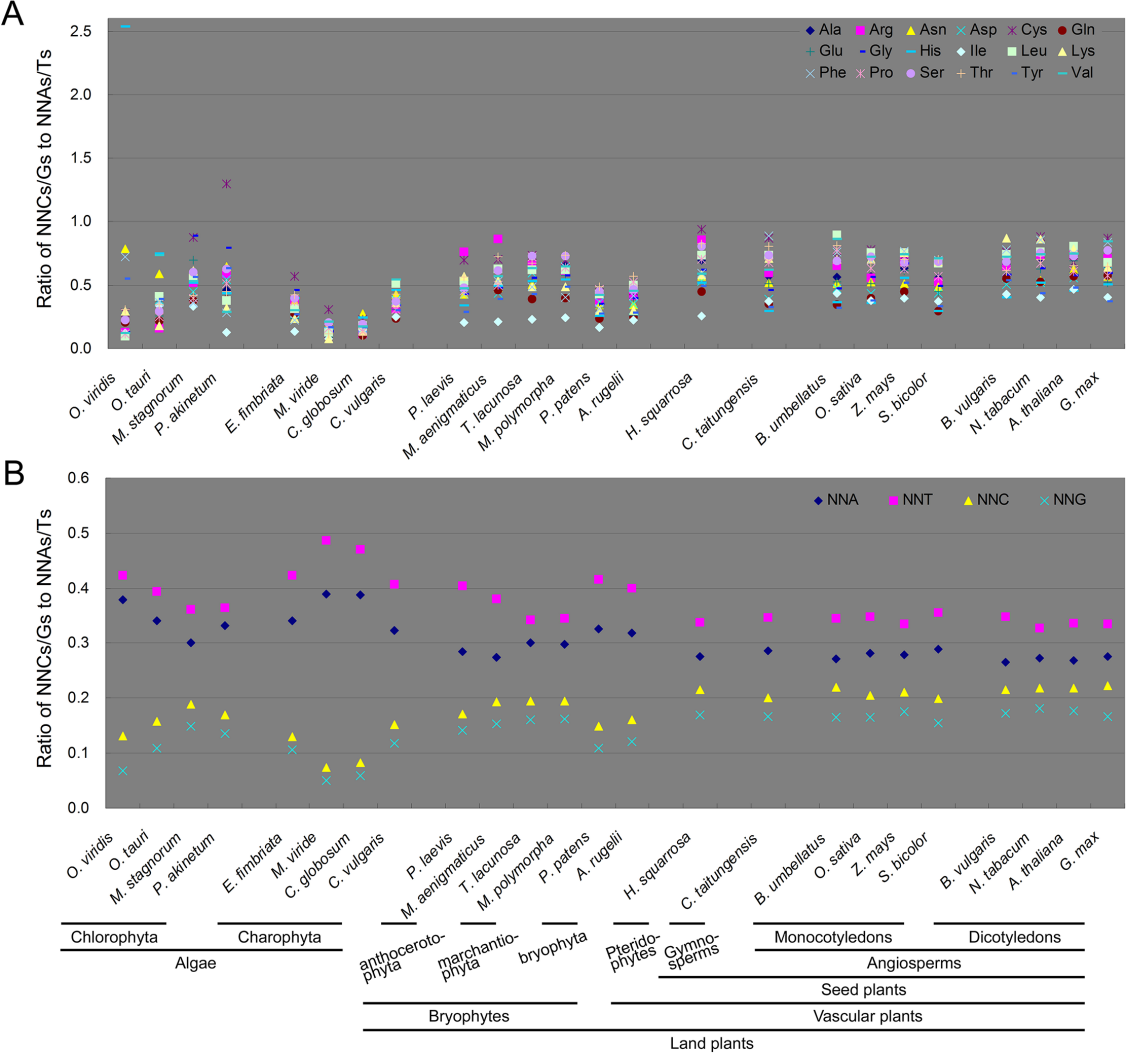
SCUB frequency in the mitochondrial genome. A. The ratio between the frequencies of NNCs/Gs and NNAs/Ts for each of 18 amino acids (Met and Trp not included). NNCs/Gs: the number of synonymous codons (SCs) as C or G as their final bases; NNAs/Ts: the number of SCs as A or T as their final base. N denotes any base. B. The frequencies of NNA, NNT, NNC and NNG. The frequency is defined as the ratio between the number of all SCs with A, T, C or G as the final base and the full set of 61 codons except for three stop codons.

We found that among the 61 codons, A- and T-ending codons (NNAs and NNTs) are more frequent than C- and G-ending codons (NNCs and NNGs) in plant mitochondrial genes (S1 Fig). In this study, to gain a direct view of SCUB, the present definition of SCUB frequency of a given amino acid encoded by synonymous codons (SCs) was the ratio of NNCs and NNGs (NNCs/Gs) number to NNAs/Ts number (Fig 1A). Generally, except for His in *O*. *viridis* and Cys in *P*. *akinetum*, the SCUB frequencies of all amino acids in 24 selected species were less than 1. In the chlorophytes, both the highest (2.536) and lowest (0.098) SCUB frequencies were identified in *O*. *Viridis*, which made its coefficient of variation (CV) markedly higher than others (1.409 vs 0.303–0.499) (S3 Table). In the charophytas, SCUB frequencies (the mean values 0.150–0.366) was significantly lower than those in chlorophytes and land plants (Fig 1A; S2A Fig). The highest frequencies represented in the charophytas were substantially lower than those present in any of the other species except for the bryophyta species (*P*. *patens* and *A*. *rugelii*). In the bryophytes, the lowest SCUB frequencies were similar; the highest values laid in the range 0.733–0.865 in the anthocerotophytes and marchantiophytes, but 0.488–0.569 in the bryophyta species (Fig 1A). In the vascular plants, SCUB frequencies were quite similar (Fig 1A; S2A Fig). The greatest range was 0.683 in *H*. *squarrosa* (pteridophyte), followed by 0.593 in *C*. *taitungensis* (gymnosperm), 0.354–0.571 among the monocotyledons and 0.354–0.495 among the dicotyledons. In total, the SCs of 18 amino acids showed preferential A- and T-ending codons, and their SCUB frequencies were significantly lower than 1 (S3 Table).

SCUB in the mitochondrial genome was directly represented by the total SCUB frequencies of NNA, NNT, NNC and NNG, which are respectively defined as the ratios of the numbers of all NNAs, NNTs, NNCs and NNGs to the codon number of all CDS in a mitochondrial genome. Here, NNA and NNT were found to be more common than NNC and NNG (Fig 1B). The frequency of NNA and NNT was significantly higher than that of NNC and NNG (*P* values were all less than 4.27E-107) (S3 Table). Of them, NNT frequency was the highest while NNG frequency the lowest (Fig 1B). In the chlorophytes, each of NNA, NNT, NNC and NNG frequencies differed between species. In *O*. *viridis*, NNA and NNT frequencies were both around 0.4, while those of NNC and NNG were around 0.1; in *M*. *stagnorum*, the frequencies of NNA, NNT and NNC, NNG were nearer to 0.3 and 0.2, respectively. In the chlorophytes, there had a clear difference in NNA and NNT from NNC and NNG frequencies. Both NNA and NNT frequencies were <0.1 in *M*. *viride* and *C*. *globosum*, while those of NNC and NNG were >0.4. In the bryophytes, the difference in the frequencies of NNA and NNT to those of NNC and NNG were quite distinctive among the anthocerotophytes, marchantiophytes and bryophyta species (Fig 1B). Of them, the preference to NNA and NNT was the weakest in marchantiophytes but the strongest in the bryophyta species. In the vascular plants, the NNA and NNT frequencies are around 0.3 and those of NNC and NNG around 0.2 (Fig 1B). The ratios of NNC/G to NNA/T in the vascular plants were higher than those in the algae and bryophytes (S2B Fig), showing their preference to NNA and NNT was weakened. These results indicate that SCUB is preferential to NNA and NNT with differential extent in different taxonomies of plants. Moreover, the ratios of C and G to A and T in the gene body (The exception was *B*. *umbellatus*), whole genome, and intron (The exception was two monocotyledonous and three dicotyledonous species) were lower than 1 (S4 Table). However, these ratios were significantly higher than the ratio of NNC/G to NNA/T in each of detected species (S4 Table). This shows that the bias to A- and T-ending codons is not absolutely due to the bias to A and T in the mitochondrial genome.

### A- and T-ending codons are more pronounced in intron-bearing genes

**Fig 2:**
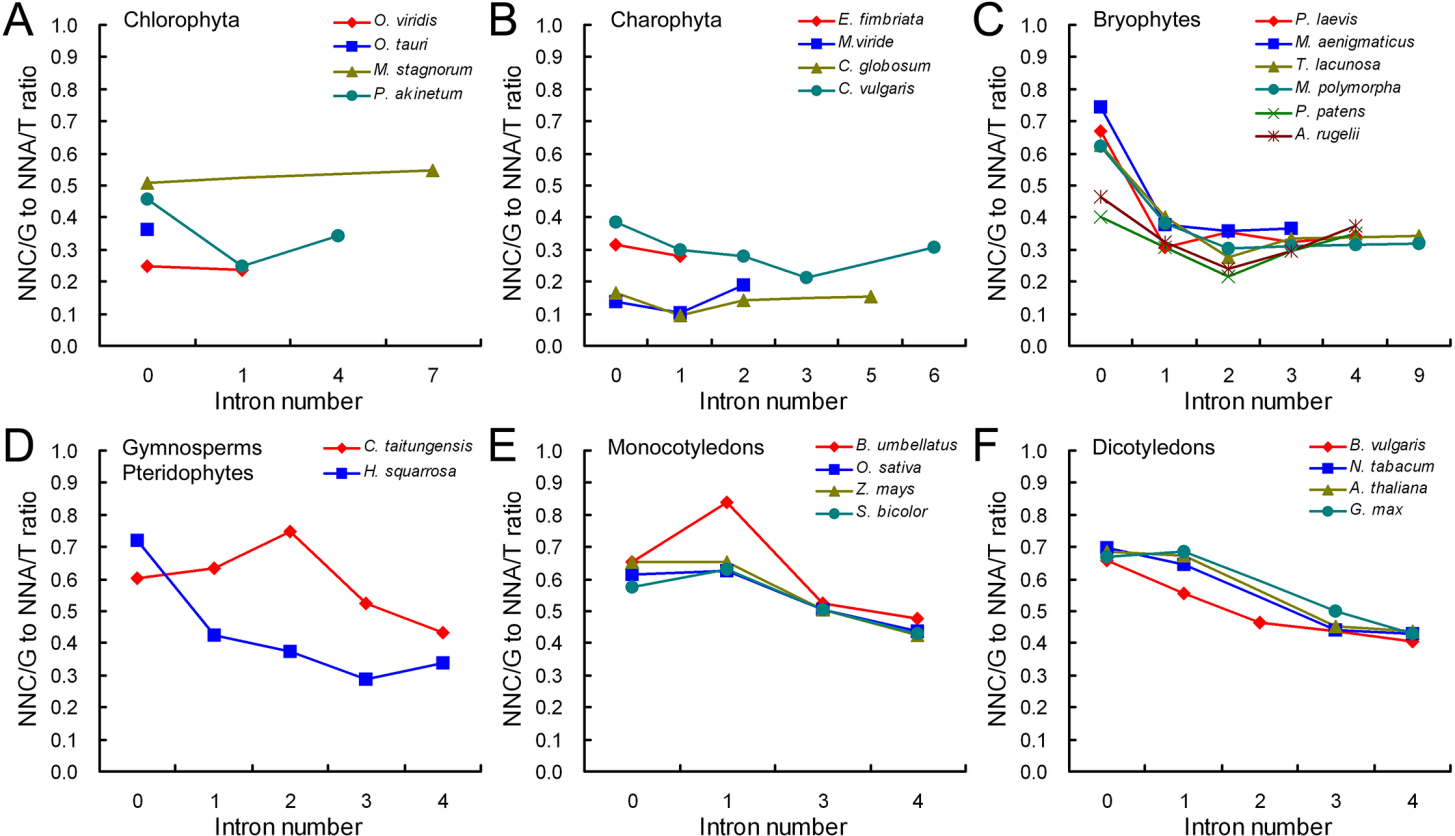
The relationship of SCUB to intron number. NNC/G to NNA/T ratio is defined as the ratio between the number of all SCs with C or G as the final base to the number of all SCs as A or T as the final base. N denotes any base.

SCUB frequency is differential in genes possessing various introns in nuclear genomes [14]. To analyze whether the characteristic was present in the mitochondrial genome, we compared SCUB frequencies among genes with various introns. The difference of SCUB frequency in genes bearing various numbers of introns was visualized by the ratio of the number of NNC and NNG (NNC/G) to that of NNA and NNT (NNA/T) (Fig 2). In the chlorophytas, intron-less genes had the higher ratio than intron-bearing genes in *P*. *akinetum* (*P* = 3.21E-14), but no difference was found among genes with various introns in other detect species (*P* > 0.4) (Fig 2A; S5 Table). Similarly, for the charophytas, the difference based on intron number was only detected in *C*. *vulgaris* (*P* = 6.48E-07) (Fig 2B; S5 Table). Unlike the algae (chlorophytas and charophytas), the ratios among gene with various introns were all significantly difference in land plants (Fig 2C–2F; S5 Table). In the bryophytes, for the anthocerotophytes and marchantiophytes, the genes without introns had easily the highest ratio, while the ratios among intron-bearing genes are comparable. For the bryophyta species, the ratios of intron-less genes were smaller than those of intron-less gene in anthocerotophytes and marchantiophytes; the ratio fell as intron number increases from zero to two, and raised when the number was larger than two (Fig 2C). In the genes of the pteridophyte *H*. *squarrosa*, the ratio fell as the intron number raised (except in the step from three introns to four) (Fig 2D). For the genes of the gymnosperm *C*. *taitungensis*, the ratio increased as the intron number raised from zero to two, but fell as it increased further to four (Fig 2D). In the genes of the angiosperms (monocotyledons and dicotyledons), the ratio decreased as the intron number raised (Fig 2E and 2F). The exception was *B*. *umbellatus*, in which the ratio was greater in genes having one intron than no introns, but fell thereafter (Fig 2E). A calculation of the mean SCUB frequency for 18 amino acids confirmed the existence of an association between SCUB and intron number (data not shown). The implication is that a preference for NNA and NNT is associated with intron number in the plant mitochondrial genome.

### SCUB is not heterogeneous with respect to the location of exons

**Fig 3:**
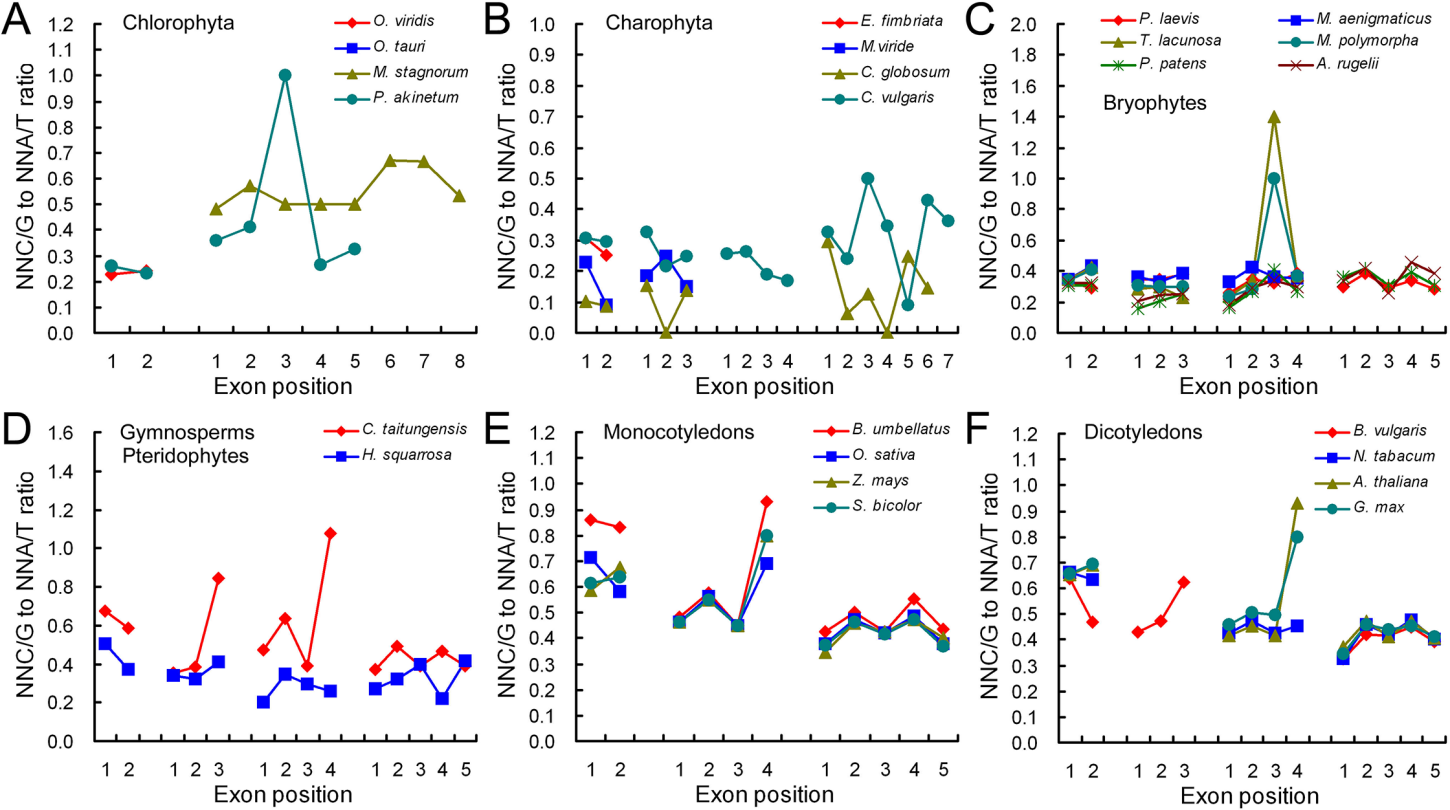
The relationship between the SCUB and exon position. NNC/G to NNA/T ratio is defined as the ratio between the number of all SCs with C or G as the final base to the number of all SCs as A or T as the final base. N denotes any base.

SCUB frequency is different among exons in nuclear genes [14], so we further analyze this rule in the mitochondrial genome using the NNC/G to NNA/T ratio. In the chlorophytes and charophytes, the ratios of exons in genes with more than two exons were around 0.2–0.3, but the patterns appeared diverse and less predictable (Fig 2A and 2B). In the bryophytes, the ratios of exons fluctuated around 0.4; the patterns were similar except that there had a peak at the third exon in genes with four exons in marchantiophytes (*T*. *lacunose*, *M*. *polymorpha*) (Fig 3C). The ratios were about 0.3–0.5 in the pteridophyte *H*. *squarrosa* and increased to 0.4–0.9 in spermatophytes (gymnosperms, monocotyledons, and dicotyledons), and the patterns were also almost identical (Fig 3D–3F). In general, the curves showed heterogeneous architectures among genes with various exons as well as in various evolutionary taxonomic plants. However, except for a few genes whose exons had obviously different SCUB frequencies (*P* < 0.05, unlined values), most genes exhibited similar SCUB frequency among exons (*P* > 0.05) (S5 Table), demonstrating that a bias towards NNA and NNT is not associated with exon position in the mitochondrial genome.

### The possible association between DNA methylation and SCUB

**Fig 4:**
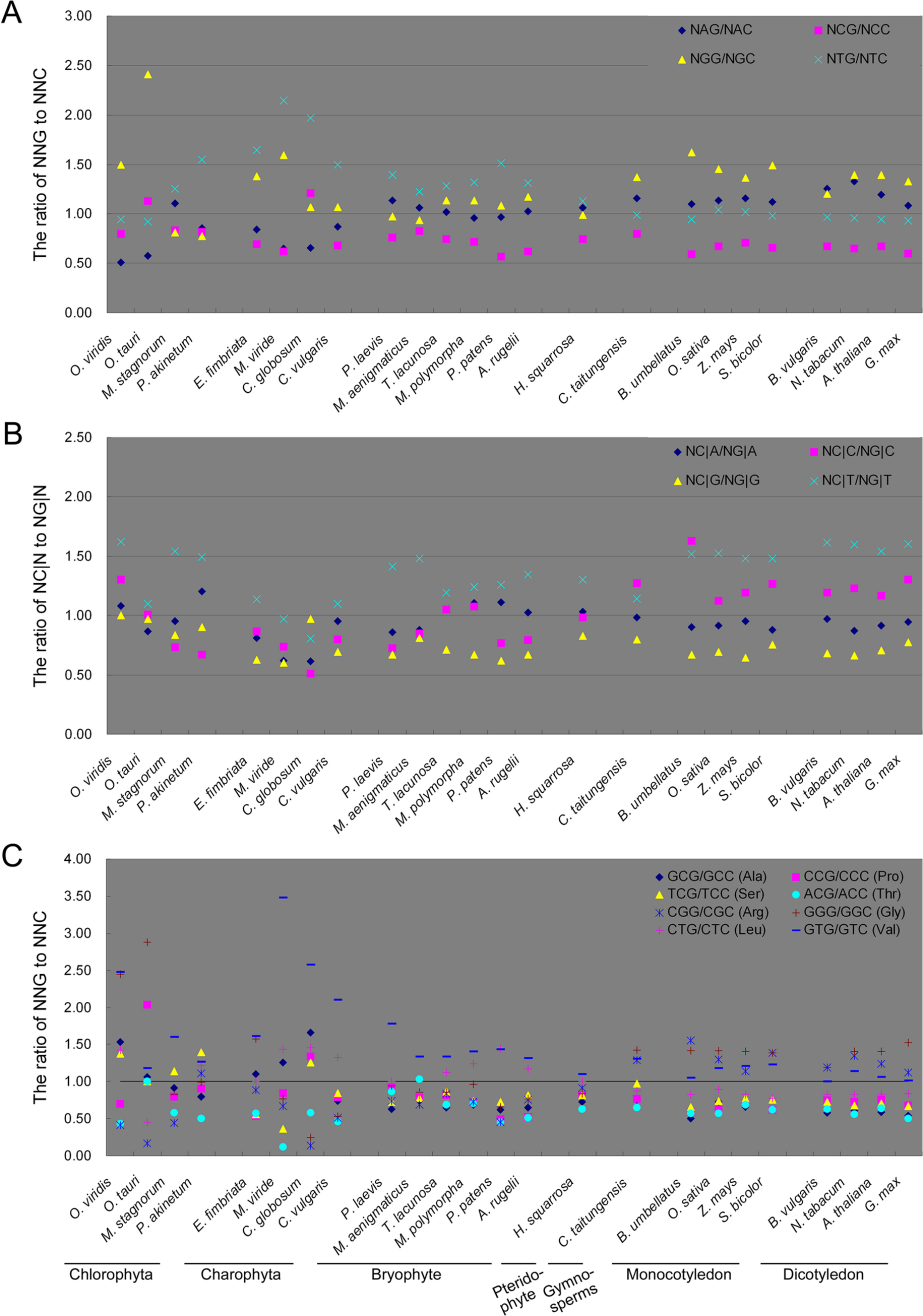
The association between the DNA methylation induced conversion of C to T and SCUB frequency. A: The ratio of the number of NNG to that of NNC based on each of four nucleotides (A, T, C, G) at the second position. B: The ratio of the number of NC|N to that of NG|N based on each of four nucleotides (A, T, G, G) at the first position of the next codon. C: The ratio of the number of NNG to that of NNC in a given amino acid.

DNA methylation is a major source of DNA variation in the nuclear genome, given that methylated cytosine (5^m^C) is readily converted into thymine [33]. The conversion of 5^m^C in CpG, or it’s complement strand, produces TpG or CpA, and the conversion of two 5^m^Cs produces TpA. Given the lower selection pressure on the third position of codons, the conversion of NCG to NCA (the second-third position) as well as NC|G to NT|G (the third-next codon’s first position) would be dominant. Our previous study showed that CpG methylation is a driver of bias to A- and T-ending SCs in the nuclear genome of land plants [14]. Unlike the nuclear genome, a set of reports found that both mitochondrial and plastid genomes are often suffered N^6^-methyladenine (m^6^A) modification but rarely experience CpG methylation in higher plants [34]. However, different amounts of 5^m^C by CpG methylation were observed in mitochondrial DNA of mammals and plants [35–40], indicating that epigenetic modifications of cytosines in the mitochondrial DNA are likely much more frequent than previously believed [39–41]. We recently found that the conversion of C to T by CpG methylation is associated with the bias to A- and T-ending SCs in plastid genomes of the vascular plants [15]. To investigate this possible association in mitochondrial genes, we compared the influence of the second-position nucleotides as well as the first nucleotides of the next codons on the frequencies of SCs with C and G at the third position. Our results show the influence of the adjacent nucleotides on the third nucleotide was diverse (Fig 4). Alike plastid genes [15], in the mitochondrial genes of land plants, the ratios of NCG/NCC were significantly lower than 1.0, but the ratios of NGG/NGC, NAG/NAC and NTG/NTC were near to or more than 1.0 (Fig 4A). In each of the vascular plants, the ratio of NCG/NCC was significantly lower compared with the penultimate (NGG/NGC in the pteridophyte *H*. *squarrosa*; NTG/NTC in seed plants) (*P* < 0.05) (S6 Table). Similarly, the ratios of NC|G/NG|G were lower compared with those of other NC|N/NG|N forms (Fig 4B), and their difference from the penultimate (NC|C/NG|C in the pteridophyte *H*. *squarrosa*; NC|A/NG|A in seed plants) was statistically significant (*P* < 0.05) (S6 Table). Unlike plastid genes [15], the association was also found in the mitochondrial genes of bryophytes. The ratios of both NCG/NCC and NC|G/NG|G were significantly lower than the penultimate in marchantiophyta and bryophyta species (*P* < 0.05), but was not in anthocerotophyta species (*P* < 0.05; the exception was NCG/NCC in *P*. *laevis*, *P* = 0.0364) (Fig 4A and 4B; S6 Table). Similar to the plastid genome [15], the association was not found in the algae. For the second-third position, three species had the lowest ratio values of NCG/NCC and NC|G/NG|G; the significant difference between NCG/NCC ratio and the penultimate was only found in *C*. *vulgaris* (*P* = 0.0148), and the significant difference between NC|G/NG|G and the penultimate was only found in *E*. *fimbriata* (Fig 4A and 4B; S6 Table).

We selected C- and G-ending SC pairs with the same nucleotides at the first and second positions within Ala, Arg, Gly, Leu, Pro, Ser, Thr and Val to further analyze the effect of the second-position nucleotide on the frequencies of C- and G- ending SCs (Fig 4C). In the vascular plants, the ratios of NCG/NCC in Ala, Pro, Ser, Thr were all less than 1, and those of NXG/NXC (X is G or C) of Arg, Gly, Leu and Val were greater than or near 1; the difference between two ratio sets were statistically significant (*P* < 0.05) (S7 Table). Inconsistent with the comparison based on SCUB frequency (Fig 4A), in the bryophytes, although NCG/NCC ratio was smaller than NXG/NXC ratio in each species (Fig 4C), the significant difference was only found in *M*. *polymorpha* (S7 Table). Oppositely, consistent with the result based on SCUB frequency, there had no significant difference between NCG/NCC ratios and NXG/NXC ratios in algae (Fig 4C; S7 Table). These results indicate that DNA methylation is possibly associated with SCUB formation in the mitochondrial genome of vascular plants, but the association in the bryophytes is weaker.

### The relationship of SCUB with phylogeny

**Fig 5:**
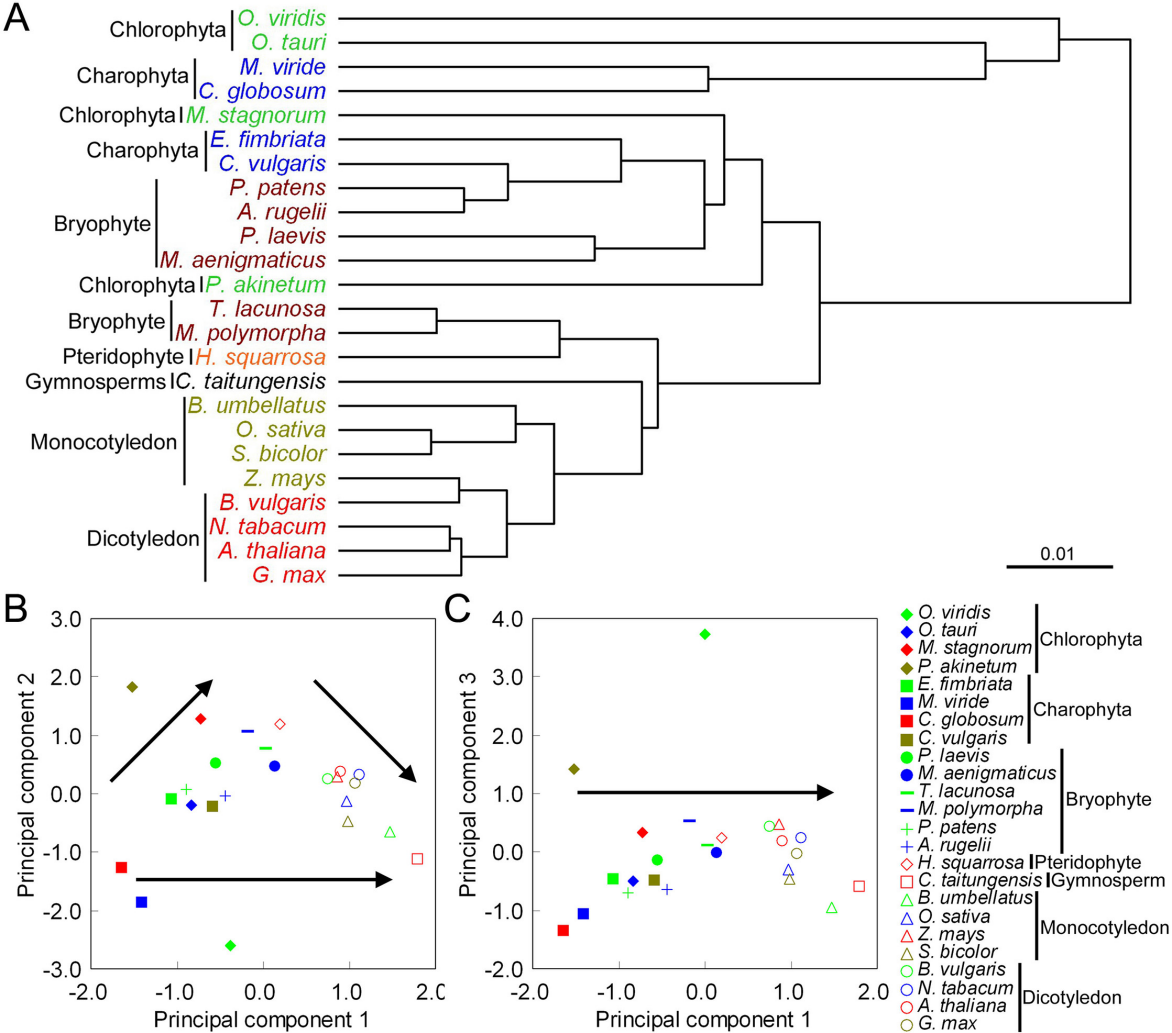
Phylogenic tree and principal component analysis of SCUB frequencies. A: the phylogenic tree based on the normalized SCUB frequencies of 59 SCs. B-C: the two-dimensional scatter plots using PC1-PC2 (B) and PC1-PC3 (C) coefficients, respectively. SCUB frequency are defined as the number of each SC to that number of total SCs.

To highlight the difference of SCUB frequency based on evolutionary taxonomy, we constructed a phylogenic tree based on the normalized SCUB frequencies of 59 codons encoding 18 amino acids (Fig 5A). In the two major clusters, two chlorophytas and two charophytas were distinct from other species, highlighting the diverse SCUB patterns in algae. In the below cluster, there had two clades that clearly distinguished the algae from the vascular plants. The bryophytes were classified into these two clades, of which anthocerotophytes and bryophyta species were close to the algae while marchantiophytes (*T*. *lacunose*, *M*. *polymorpha*) were near to the vascular plants, demonstrating the position of bryophytes during the evolution from algae to land plants. In the clade with land plants, the species were subdivided into two groups, one containing marchantiophytes and the pteridophyte *H*. *squarrosa*, and the other including spermatophytes where monocotyledons and dicotyledons exhibited closer phylogenic relationships.

The principal component analysis (PCA) further highlighted the evolutionary taxonomic difference across plant species (Fig 5B and 5C). The first PC (PC1) grouped the chlorophytes with the charophytes, and somewhat separates these from the bryophytes (Fig 5B). Among the bryophytes, *P*. *patens* and *A*. *rugelii* clusters with the algal species, while the marchantiophytes are separated from the non-land plants. The pteridophyte *H*. *squarrosa* is rather distinct from the other lower plants. Surprisingly, the angiosperms appear to lie rather closer to the lower plants than the gymnosperm *C*. *taitungensis*. PC2 separates the three categories of the bryophytes from one another as well as monocotyledons from dicotyledons, while PC3 distinguishes the chlorophytes from the charophytes (Fig 5B and 5C). The PCA recreates the evolutionary difference from lower to higher plants in both PC1-PC2 and PC1-PC3 spaces. Specifically, in PC1-PC2 space, the evolution from the algae via three categories of bryophytes to the vascular plants is readily visualized (see the up arrow), while that from lower to higher embryophytes is illustrated by the down arrow (Fig 5B). Together, SCUB in mitochondrial genomes can visualize difference of plant evolution.

To further evaluate the specific contributions of each codon on these three PCs, their correlation coefficients (absolute value > 0.6) with the PCs were selected [42]. In total, there had 45 codons whose correlation coefficients > 0.6 with either of three PCs (S8 Table). The codons with negative correlation to PCs (correlation coefficient < -0.6) were those with A and T at the third position; on the contrary, the codons with positive correlation to PCs (correlation coefficient > 0.6) were those ended with C and G (The exceptions were CCA and AGA in PC1). Moreover, the second position also affected the correlation coefficients of codons. For PC1, codons with correlation coefficients < -0.6 were characterized by A or T at the second position (seven out of nine), but those with C or G at the second position were dominant among codons with correlation coefficients > 0.6 (thirteen out of nineteen). For PC2, the second nucleotide of both positively and negatively correlative codons was mostly C or G. For PC3, all of four correlative codons had A at the second position. Interestingly, the start and stop codons had no obvious contribution to these PCs.

## Discussion

### SCUB in mitochondrial genes targets NNA and NNT codons

The ancestors of land-based plants are presumed to be single celled algae. The prolonged period of selection experienced by algae species has tended to favour GC enrichment in their nuclear genomes [43]. Among the land plants, some species show a bias for NNA or NNT SCs, while others target NNC and NNG (Campbell and Gowri, 1990; Tatarinova et al., 2010; Qin et al., 2013). Here, we have shown that SCUB in the mitochondrial genome is focused on NNA and NNT rather than on NNC and NNG in both the algae and the land plants (Fig 1; S3 Table). With respect to the stop codons, TAA and TGA tend to be favoured over TAG, and the bias to NNA and NNT is also present in the internal stop codons (Table 2; S2 and S9 Tables). The usage of codons is majorly determined by the GC content of genes; G/C-ending codons are more preferential in GC-richer genes or genomes, and vice versa [44]. Consistently, strong correlations are present between the ratio of NNC/G to NNA/T and the ratios of GC to AT of gene body, genome sequence, intron sequence, respectively (*R* = 0.879, 0.852, 0.732, respectively). Thus, it could be suggested that the preference to A-/T-ending codons is partially due to the AT richness in plant mitochondrial genomes. Note that the ratios of NNC/G to NNA/T are significantly lower than the ratios of C and G to A and T in the gene body, intron and whole genome sequences (S4 Table). This indicates a bias towards A and T of SCs is not absolutely under a neutral or mutational model. On the other hand, DNA recombination and indels induce a range of genomic shock associated events such as single nucleotide change [45]. Single nucleotide change appears to be heavily biased towards A and T [8]. Both sequence deletion in the mitochondrial genome, as well as its transfer into the nuclear genome, is typically followed by recombination and the formation of indels, and induces signal nucleotide change [45], so the outcome has been an increased frequency in A and T-ending SCs. Unlike the lower plants, the preference to NAA and NNT codons is uniform in the vascular plants (Fig 1; S2 Fig), suggesting that the evolution pattern and selection pressure of SCs appear to be more similar in higher plants. With respect to the less common start codons, ACG is apparently preferred over NNC and other NNG codons (Table 2). A possible explanation for this bias is that mitochondrial mRNA often experiences cytosine deamination post-transcriptionally, which results in its conversion to uridine [46], therefore changing the ACG codon into the canonical start codon AUG. Since ACG is found as a start codon only in the land plants (Table 2), it is feasible that the conversion of cytosine to uracil by deamination evolved only after the appearance of the land plants.

### SCUB of mitochondrial genes differentiates plants of different evolutionary taxonomies

The accepted chronology of plant evolution leads from the algae, via the bryophytes and pteridophytes, to the gymnosperms and angiosperms. Our previous work showed that SCUB in both the nuclear and plastid genomes mirrors the evolution of plants [14, 15]. The present analysis of mitochrondial gene sequences has demonstrated that the preference for NNA and NNT varies across the range of species examined (Fig 1; S2 Fig; S3 Table). This variation mirrors the difference in the evolutionary taxonomy of the plant kingdom (Fig 5). Interestingly, PCA analysis shows that the bryophytes and pteridophytes are separated by the spermatophytes (Fig 5B). This does not coincide with the evolutionary levels of these taxonomic clades of plants, suggesting asynchronous evolutionary behaviours between the mitochondrial genomes and plants. The speculation is that mitochondrial genomes of these plants are under varied selection pressures, because SCUB is an indicator for reflecting balance between mutation, genetic drift and natural selection [9, 10].

Whole-genome duplication (WGD) and polyploidization are recognized as one of the major drivers of genome evolution (Vision et al., 2000; Bowers et al., 2003; Tang et al., 2008; Barker et al., 2009). Both the divergence of spermatophytes from pteridophyte, and that of angiosperms from gymnosperms involved WDG events [45]. Our previous analysis of SCUB in the nuclear genome demonstrated that only those polyploidization events which occurred post the appearance of the angiosperms had any influence on SCUB [14]. Looking at the perspective of the mitochrondial genome, the spermatophytes appear distinct from the other plant groups, the gymnosperms are not clustered with the angiosperms, nor the monocotyledons with the dicotyledons (Fig 4A), suggesting that both WGD and polyploidization have made a contribution to SCUB in the mitochondrial genome. In line with the similar rule in the plastid genome [15], the implication is that, unlike the nuclear genome, these evolutionary events have no association with the SCUB of the organellar genomes.

### Intron evolution is a driver of SCUB in the mitochondrial genome

Intron evolution is a major evolutionary event in eukaryotic genomes [47]. It is frequently associated with the induction of sequence alterations in the adjacent exons, forming either SCs or non-synonymous codons that lead to a bias towards lower GC content [48]. We previously found that the frequency of NNA and NNT SCs rises as the intron number increases in the nuclear genes [14], but this rule is not present in the plastid genome [15]. Here, SCUB is also associated with intron number in the mitochondrial genomes of land plants, and the frequency of NAA and NNT SCs is generally correlated with the intron number; while in the algae, the correlation is much unobvious (Fig 2; S5 Table). Genes harbouring fewer introns are thought to be both favoured by selection and to evolve slowly [49], with the result that the GC content of the exonic fraction has tended to rise over time [50]. This indicates genes with more introns would have less selection pressure to encode functional proteins, so they should have stronger bias to A/T-ending codons, which are originated from the mutation pressure [8]. Thus, unlike the plastid genome, SCUB favours GC in genes with fewer introns in the mitochondrial genome (Fig 2; S5 Table), showing the difference in the association between SCUB and intron evolution in the organellar genomes. Note that, in the mitochondrial genomes of some species, genes harbouring one or two introns are more preferential to NAA or NNT SCs than intronless genes (Fig 2), which is of some interest to be further studied.

The gain/loss of an intron only induces nucleotide change in the flanking exons, because indels can lead to base changes over a distance of several hundred bases [7, 51]. Our previous work showed that in the nuclear genome, interstitial exons favour NNA and NNT SCs, while the terminal ones (especially the most 5’ ones) prefer NNC and NNG [14], but the preference is absent in the plastid genome [15]. In the mitochrondrial genome, SCUB frequency is equally distributed among the exons, although the pattern looks heterogeneous both with respect to exon position and the evolutionary status of the species (Fig 3; S5 Table). Exons showing bias towards NNC or NNG SCs are less affected by intron evolution [52, 53], so intron evolution is concentrated in the interstitial part (exons) in the nuclear genome [14]. Indels are concentrated in GC poor regions [51, 54], and that their effect is to further reduce GC content [48]. In combination with the findings of the plastid and mitochondrial genomes, it seems possible that intron gain/loss in the organellar genome has similar preference in the terminal rather and interstitial exons, in contrast to the situation prevailing in the nuclear genome [14].

### DNA methylation is possibly associated with SCUB formation in mitochondrial genomes

The DNA methylation induced conversion of C to T is a potent agent of naturally occurring mutagenesis [55]. Our previous study showed that in land plants, the bias towards NNA and NNT in the nuclear genome is positively correlated with DNA methylation-mediated conversion of C to T [14]. Although 5^m^C by CpG DNA methylation rarely experiences in the plastid genome [56, 57], such association based on 5^m^C was found in the vascular plants [15]. Consistent with the plastid genome, DNA methylation-mediated conversion has a strong association with SCUB of mitochondrial genome in the vascular plants; while unlike the plastid genome, the association is also present in some species of bryophytes (Fig 4; S6 and S7 Tables). These results indicate that DNA methylation accounts for SCUB in both nuclear and organellar genomes of land plants (vascular plants and some bryophytes). In addition, DNA methylation is a major driver of SCUB during intron evolution of the land plants [14]. A CpG under-representation in mitochondrial DNA has always suggested a susceptibility to mutation of this dinucleotide also in the mitochondrial genome and, consequently, to methylation [36, 58, 59]. Thus, unlike the nuclear and plastid genomes whose DNA methylation-induced C-to-T convertion appeared during the evolution from algae to land plants and from bryophytes to vascular plants, respectively, the contribution of DNA methylation in SCUB in mitochondrial genomes possibly appeared in the divergence of bryophytes, so that its effect was detected in some species of bryophytes and all vascular plants.

In the nuclear genome, DNA methylation is an ancient property of nuclear genomes from algae to land plants [60], but DNA methylation-induced SCUB is present in land plants [14], showing the DNA methylation-induced nucleotide substitution is possibly an evolutionary event independent of DNA-methylation. Note that although some reports indicating the existence of CpG methylation in mitochondrial DNA of mammals and plants [35–40], the view that this DNA modification is rare in the organellar genomes is prevailing [56, 57]. One implication is that DNA methylation-induced SCUB is possibly a consequence and trace of a previous evolution event (DNA methylation) that had lost during the following plant evolution. This possible event is similar to the evolution of internal stop codons in plastid genes. Internal stop codons are present in the available plastid genomes of most leptosporangiate ferns, but not in either most early diverging fern lineages or seed plants [32], which is resulted from the fact that U-to-C editing originated in the common ancestor of vascular plants and hornworts, with independent losses from the lycophyte Selaginella and most (or all) seed plants [29]. Together, DNA-methylation induced SCUB in the evolution and divergence of mitochondria is an important topic that needs to be addressed. These data also provide an evidence for further understand why dinucleotide frequency can act as a signature of genomic heterogeneity (Karlin, 1998). Moreover, mutational patterns, translational selection, translational accuracy, mRNA stability, protein stability, and interference selection are also drivers of SCUB [8]. The association between these forces and DNA methylation in SCUB needs to be investigated further.

## Supporting Information

S1 Table The CDS sequences extracted from 24 selected plant mitochondrial genomes. (XLS)

S2 Table The distribution of internal stop codons. (PDF)

S3 Table The statistical analysis of SCUB frequencies. SCUB frequencies based on amino acids defined as the difference in the SCUB frequencies (the ratios of C-/G-ending SCs (NNCs/Gs) to NNAs/Ts) of 18 amino acids and 1 using the one-sample *t*-test. SCUB based on NNA/T and NNC/G defined as the difference between the frequency of all C-/G-ending codons (NNC/G) to NNA/T using the numbers of NNC/G and NNA/T with the chi square (χ^2^) test.
(PDF)

S4 Table The comparison of the ratios of NNC/NNG to NNA/NNT with the ratios of C and G to A and T in the gene body, intron and whole genome sequences. (a) The difference in C and G from A and T in the gene body, intron, and whole genome sequences is calculated with the chi square (χ ^2^) test. (b) The difference in the ratio of NNC/G to NNA/T from the ratios of C and G to A and T in the gene body, intron, and whole genome sequences is calculated by the chi square (χ ^2^) test of the cross-table analysis.
(PDF)

S5 Table The statistical analysis of SCUB frequency based on intron number and exon position. Intron number: the comparison of SCUB frequencies among genes bearing various amounts of introns using the numbers of NNC/G and NNA/T. Exon position: the comparison of SCUB frequencies among exons in genes using the numbers of NNC/G and NNA/T. The difference significance is calculated by the chi square (χ ^2^) test of the cross-table analysis
(PDF)

S6 Table The statistical analysis of the association between the DNA methylation induced conversion of C to T and SCUB frequency. The numbers of NCG, NCC and other NNN combinations are used for analysis with the chi square (χ ^2^) test of the cross-table analysis.
(PDF)

S7 Table The statistical analysis of the association between the DNA methylation induced conversion of C to T and SCUB frequency based on special amino acids. The ratios of NCG/NCC of of Ala, Pro, Ser, Thr as well as the ratios of NXG/NXC (X is G or C) of Arg, Gly, Leu and Val are used for the Mann-Whitney test.
(PDF)

S8 Table The correlation coefficients of codons to principal components. The absolute values greater than 0.6 were selected.
(PDF)

S9 Table The statistical analysis of usage bias of stop codons and internal stop codons. The bias to A-ending stop codons and internal stop codons was analyzed using the numbers of TAA+TGA and TAG with the chi square (χ^2^) test.
(PDF)

S1 Fig The frequency of 61 amino acid encoding codons in chloroplast genomes. The index is defined as the number of each codon to the number of total 61 codons.
(PDF)

S2 Fig The statistical analysis of SCUB frequencies among the algae, bryophytes, pteridophytes, gymnosperms, monocotyledons and dicotyledons. The ratios of NNCs/Gs to NNAs/Ts of 18 amino acids (A) and the ratios of NNC/G to NNA/T (B) are used for analysis with the stepwise comparison of Kruskal-Wallis test. The data are presented as the box plot of the ratios of different species. The boxes without the same lowercase letter mean significantly different from each other.
(PDF)
