# Supplementary material for: Synonymous Codon Usage Bias in Plant Mitochondrial Genes Is Associated with Intron Number and Mirrors Species Evolution
Source: PLoS One. 2015 Jun 25;10(6):e0131508. doi: 10.1371/journal.pone.0131508 (PMC4481540; doi:10.1371/journal.pone.0131508)
Supplement: S2 Table — (PDF) [file pone.0131508.s002.pdf]

S2 Table. The distribution of internal stop codons

Species	Codon	Number of introns in genes					Total
		0	1	2	3	4	
P. laevis	TAA	8	3	14	13	10	48
	TGA	6		6	7	5	24
	TAG	2			4	2	8
M. aenigmaticus	TAA	9	2	10	5		26
	TGA	4		5	5		14
	TAG	1		1	3		5
T. lacunosa	TAA	1					1
	TGA						
	TAG						
H. squarrosa	TAA	1				2	3
	TGA						0
	TAG			1			1
