# Supplementary material for: Synonymous Codon Usage Bias in Plant Mitochondrial Genes Is Associated with Intron Number and Mirrors Species Evolution
Source: PLoS One. 2015 Jun 25;10(6):e0131508. doi: 10.1371/journal.pone.0131508 (PMC4481540; doi:10.1371/journal.pone.0131508)
Supplement: S3 Table — SCUB frequencies based on amino acids defined as the difference in the SCUB frequencies (the ratios of C-/G-ending SCs (NNCs/Gs) to NNAs/Ts) of 18 amino acids and 1 using the one-sample t-test. SCUB based on NNA/T and NNC/G defined as the difference between the frequency of all C-/G-ending codons (NNC/G) to NNA/T using the numbers of NNC/G and NNA/T with the chi square (χ2) test. (PDF) [file pone.0131508.s003.pdf]

S3 Table. The statistic analysis of SCUB frequencies

Taxomony	Species	SCUB based on amino acids			SCUB based on NNA/T and NNC/G		
		Mean	CV	*P* value	NNA/T	NNC/G	*P* value
Chlorophyta	*O. viridis*	0.405	1.409	3.68E-04	7747	1914	0
	O. tauri	0.367	0.467	1.55E-11	7105	2575	0
	M. stagnorum	0.537	0.303	8.84E-10	9209	4695	0
	P. akinetum	0.508	0.499	2.47E-07	10708	4683	0
Charophyta	*E. fimbriata*	0.332	0.307	1.26E-15	6726	2086	0
	M. viride	0.150	0.387	1.77E-21	7713	1095	0
	C. globosum	0.168	0.272	4.53E-23	8590	1408	0
	C. vulgaris	0.366	0.226	9.78E-17	9159	3388	0
Bryophyte	*P. laevis*	0.477	0.274	4.22E-12	5234	2380	1.22E-234
	M. aenigmaticus	0.535	0.264	9.58E-11	5551	2930	3.61E-178
	T. lacunosa	0.565	0.234	9.66E-11	10812	5980	2.51E-304
	M. polymorpha	0.572	0.222	6.52E-11	12991	7213	0
	P. patens	0.353	0.246	1.51E-16	8401	2931	0
	A. rugelii	0.390	0.252	3.17E-15	8594	3379	0
Pteridophyte	*H. squarrosa*	0.630	0.261	3.04E-08	13791	8671	8.81E-256
Gymnosperms	*C. taitungensis*	0.579	0.326	3.43E-08	6990	4051	3.75E-172
Monocotyledon	*B. umbellatus*	0.604	0.302	5.04E-08	5560	3471	4.27E-107
	O. sativa	0.575	0.248	4.56E-10	9220	5420	1.67E-216
	Z. mays	0.622	0.195	2.27E-10	19322	12168	0
	S. bicolor	0.535	0.279	2.23E-10	6450	3544	8.93E-186
Dicotyledon	*B. vulgaris*	0.624	0.196	2.76E-10	17373	10981	0
	N. tabacum	0.659	0.220	1.65E-08	15915	10636	2.98E-230
	A. thaliana	0.638	0.172	9.03E-11	13251	8676	1.35E-209
	G. max	0.631	0.221	2.77E-09	10777	6855	9.83E-192
