# Supplementary material for: Synonymous Codon Usage Bias in Plant Mitochondrial Genes Is Associated with Intron Number and Mirrors Species Evolution
Source: PLoS One. 2015 Jun 25;10(6):e0131508. doi: 10.1371/journal.pone.0131508 (PMC4481540; doi:10.1371/journal.pone.0131508)
Supplement: S4 Table — (a) The difference in C and G from A and T in the gene body, intron, and whole genome sequences is calculated with the chi square (χ 2) test. (b) The difference in the ratio of NNC/G to NNA/T from the ratios of C and G to A and T in the gene body, intron, and whole genome sequences is calculated by the chi square (χ 2) test of the cross-table analysis. (PDF) [file pone.0131508.s004.pdf]

S4 Table. The comparison on the ratios of NNC/NNG to NNA/NNT with the ratios of C and G to A and T in the gene body, intron, and whole genome sequences

Taxomony	Species	CDS	Gene			Genome			Intron		
		NNC/G to NNA/T	CG to AT	P value (a)	P value (b)	CG to AT	P value (a)	P value (b)	CG to AT	P value (a)	P value (b)
Chlorophyta	*O. viridis*	0.247	0.523	0.000	9.86E-163	0.501	0.000	9.56E-156	0.559	2.81E-65	7.81E-83
	O. tauri	0.362	0.592	0.000	5.65E-81	0.619	0.000	4.98E-103	-	-	-
	M. stagnorum	0.510	0.679	0.000	1.57E-47	0.692	0.000	3.33E-57	0.695	3.83E-95	5.24E-35
	P. akinetum	0.437	0.611	0.000	6.62E-71	0.648	0.000	4.19E-99	0.586	9.87E-141	2.47E-26
Charophyta	*E. fimbriata*	0.310	0.585	0.000	4.18E-118	0.745	2.15E-287	2.33E-254	0.771	2.43E-11	3.62E-88
	M. viride	0.142	0.447	0.000	2.59E-261	0.475	0.000	1.52E-304	0.409	1.09E-100	4.15E-90
	C. globosum	0.164	0.477	0.000	4.09E-282	0.525	0.000	0.000	0.522	3.78E-149	1.50E-210
	C. vulgaris	0.370	0.697	0.000	4.09E-191	0.692	0.000	2.23E-189	0.903	1.53E-13	8.41E-301
Bryophyte	*P. laevis*	0.455	0.845	2.58E-156	2.10E-133	0.805	3.11E-267	1.94E-117	0.920	2.41E-28	1.37E-168
	M. aenigmaticus	0.528	0.889	1.90E-68	6.70E-108	0.852	2.07E-258	2.18E-95	0.967	2.56E-05	1.59E-140
	T. lacunosa	0.553	0.801	7.76E-250	2.86E-101	0.766	0.000	4.20E-83	0.931	5.49E-13	1.86E-168
	M. polymorpha	0.555	0.772	0.000	7.71E-96	0.736	0.000	2.45E-75	0.912	2.47E-22	2.18E-178
	P. patens	0.349	0.694	0.000	1.47E-203	0.683	0.000	5.95E-204	0.864	9.43E-37	0.000
	A. rugelii	0.393	0.718	0.000	6.27E-173	0.701	0.000	3.76E-166	0.868	1.74E-37	1.42E-262
Pteridophyte	*H. squarrosa*	0.629	0.865	1.65E-161	4.11E-105	0.791	0.000	3.85E-60	0.976	0.001	6.76E-175
Gymnosperms	*C. taitungensis*	0.580	0.924	8.78E-27	8.15E-110	0.884	3.06E-116	2.53E-100	0.903	1.18E-95	8.74E-107

Monocotyledon	*B. umbellatus*	0.624	1.008	0.351	9.31E-95	0.965	3.00E-12	1.19E-89	1.338	3.34E-60	7.91E-165
	O. sativa	0.588	0.810	1.23E-299	4.91E-75	0.781	0.000	2.44E-60	0.831	1.34E-211	3.53E-82
	Z. mays	0.630	0.825	6.13E-249	1.34E-97	0.784	0.000	7.14E-76	0.792	0.000	3.36E-82
	S. bicolor	0.549	0.883	2.59E-48	8.40E-99	0.777	0.000	4.28E-61	1.099	1.75E-09	1.43E-156
Dicotyledon	*B. vulgaris*	0.632	0.782	0.000	7.85E-55	0.781	0.000	2.90E-63	1.027	0.163	2.14E-102
	N. tabacum	0.668	0.831	2.21E-273	8.33E-58	0.817	0.000	8.99E-55	1.044	0.004	2.55E-115
	A. thaliana	0.655	0.819	0.000	8.07E-57	0.811	5.34E-293	3.10E-51	0.820	0.000	1.80E-57
	G. max	0.636	0.876	1.50E-85	1.03E-80	0.819	3.43E-290	3.46E-58	0.987	0.351	3.89E-100
