# Supplementary material for: Synonymous Codon Usage Bias in Plant Mitochondrial Genes Is Associated with Intron Number and Mirrors Species Evolution
Source: PLoS One. 2015 Jun 25;10(6):e0131508. doi: 10.1371/journal.pone.0131508 (PMC4481540; doi:10.1371/journal.pone.0131508)
Supplement: S5 Table — Intron number: the comparison of SCUB frequencies among genes bearing various amounts of introns using the numbers of NNC/G and NNA/T. Exon position: the comparison of SCUB frequencies among exons in genes using the numbers of NNC/G and NNA/T. The difference significance is calculated by the chi square (χ 2) test of the cross-table analysis (PDF) [file pone.0131508.s005.pdf]

S5 Table. The statistical analysis of SCUB frequency based on intron number and exon position

Taxomony	Species	Intron number	Exon position							
			2-exons	3-exons	4-exons	5-exons	6-exons	7-exons	8-exons	10-exons
Chlorophyta	*O. viridis*	0.568	0.748							
	O. tauri									
	M. stagnorum	0.428							0.968	
	P. akinetum	3.21E-14	0.471			0.407				
Charophyta	*E. fimbriata*	0.167	0.221							
	M. viride	0.010	0.086	0.255						
	C. globosum	0.066	0.686	0.725			<u>0.038</u>			
	C. vulgaris	6.48E-07	0.802	0.613	0.539			0.574		
Bryophyte	*P. laevis*	7.21E-41	0.556	0.793	0.097	0.698				
	M. aenigmaticus	3.03E-52	0.458	0.469	0.339					
	T. lacunosa	1.42E-44	0.056	0.407	<u>0.023</u>					<u>4.13E-04</u>
	M. polymorpha	3.16E-50	0.072	0.960	0.076					0.044
	P. patens	1.84E-13	0.980	0.146	<u>0.030</u>	0.866				
	A. rugelii	1.33E-21	0.961	0.684	0.237	0.731				
Pteridophyte	*H. squarrosa*	2.96E-81	<u>0.009</u>	0.128	0.181	0.223				
Gymnosperms	*C. taitungensis*	1.78E-09	0.248	<u>0.006</u>	<u>0.047</u>	0.301				
Monocotyledon	*B. umbellatus*	7.67E-12	0.795		0.307	0.368				
	O. sativa	3.84E-09	<u>0.027</u>		0.635	0.365				
	Z. mays	6.13E-21	0.207		0.506	0.169				

	S. bicolor	3.71E-07	0.776		0.506	0.384
Dicotyledon	*B. vulgaris*	3.80E-20	0.059	0.696		0.418
	N. tabacum	6.93E-22	0.683		0.950	0.304
	A. thaliana	7.44E-19	0.582		0.258	0.497
	G. max	3.54E-16	0.672		0.632	0.460
