# Supplementary material for: Synonymous Codon Usage Bias in Plant Mitochondrial Genes Is Associated with Intron Number and Mirrors Species Evolution
Source: PLoS One. 2015 Jun 25;10(6):e0131508. doi: 10.1371/journal.pone.0131508 (PMC4481540; doi:10.1371/journal.pone.0131508)
Supplement: S6 Table — The numbers of NCG, NCC and other NNN combinations are used for analysis with the chi square (χ 2) test of the cross-table analysis. (PDF) [file pone.0131508.s006.pdf]

S6 Table. The statistical analysis of the association between the DNA methylation induced conversion of C to T and SCUB frequency

Taxomony	Species	The second-third nucleotides		The third and next first nucleotides	
		Combination	*P* value	Combination	*P* value
Chlorophyta	*O. viridis*	-	-	NC|G/NG|G vs NC|A/NG|A	0.5234
	O. tauri	-	-	-	-
	M. stagnorum	-	-	-	-
	P. akinetum	-	-	-	-
Charophyta	*E. fimbriata*	NCG/NCC vs NAG/NAC	0.1128	NC|G/NG|G vs NC|A/NG|A	0.0170
	M. viride	NCG/NCC vs NAG/NAC	0.7672	NC|G/NG|G vs NC|A/NG|A	0.7873
	C. globosum	-	-	-	-
	C. vulgaris	NCG/NCC vs NAG/NAC	0.0148	NC|G/NG|G vs NC|C/NG|C	0.1654
Bryophyte	*P. laevis*	NCG/NCC vs NGG/NGC	0.0364	NC|G/NG|G vs NC|C/NG|C	0.5169
	M. aenigmaticus	NCG/NCC vs NGG/NGC	0.2042	NC|G/NG|G vs NC|C/NG|C	0.6736
	T. lacunosa	NCG/NCC vs NAG/NAC	1.6954E-05	NC|G/NG|G vs NC|C/NG|C	5.9559E-08
	M. polymorpha	NCG/NCC vs NAG/NAC	6.0982E-06	NC|G/NG|G vs NC|C/NG|C	6.5892E-13
	P. patens	NCG/NCC vs NAG/NAC	8.5666E-07	NC|G/NG|G vs NC|C/NG|C	0.0460
	A. rugelii	NCG/NCC vs NAG/NAC	5.1809E-07	NC|G/NG|G vs NC|C/NG|C	0.0917
Pteridophyte	*H. squarrosa*	NCG/NCC vs NGG/NGC	2.4305E-06	NC|G/NG|G vs NC|C/NG|C	0.0047
Gymnosperms	*C. taitungensis*	NCG/NCC vs NTG/NTC	0.0046	NC|G/NG|G vs NC|A/NG|A	0.0091
Monocotyledon	*B. umbellatus*	NCG/NCC vs NTG/NTC	1.6284E-08	NC|G/NG|G vs NC|A/NG|A	0.0006
	O. sativa	NCG/NCC vs NTG/NTC	1.4451E-10	NC|G/NG|G vs NC|A/NG|A	4.3410E-05
	Z. mays	NCG/NCC vs NTG/NTC	2.4718E-14	NC|G/NG|G vs NC|A/NG|A	8.7564E-17
	S. bicolor	NCG/NCC vs NTG/NTC	2.7069E-06	NC|G/NG|G vs NC|A/NG|A	0.0758
Dicotyledon					
	B. vulgaris	NCG/NCC vs NTG/NTC	8.2092E-13	NC|G/NG|G vs NC|A/NG|A	6.7530E-13
	N. tabacum	NCG/NCC vs NTG/NTC	6.0027E-15	NC|G/NG|G vs NC|A/NG|A	1.2717E-07
	A. thaliana	NCG/NCC vs NTG/NTC	1.8486E-09	NC|G/NG|G vs NC|A/NG|A	2.8987E-06
	G. max	NCG/NCC vs NTG/NTC	1.8338E-12	NC|G/NG|G vs NC|A/NG|A	0.0013
