# Supplementary material for: Synonymous Codon Usage Bias in Plant Mitochondrial Genes Is Associated with Intron Number and Mirrors Species Evolution
Source: PLoS One. 2015 Jun 25;10(6):e0131508. doi: 10.1371/journal.pone.0131508 (PMC4481540; doi:10.1371/journal.pone.0131508)
Supplement: S7 Table — The ratios of NCG/NCC of of Ala, Pro, Ser, Thr as well as the ratios of NXG/NXC (X is G or C) of Arg, Gly, Leu and Val are used for the Mann-Whitney test. (PDF) [file pone.0131508.s007.pdf]

S7 Table. The statistical analysis of the association between the DNA methylation induced conversion of C to T and SCUB frequency based on special amino acids

Taxomony	Species	Means of NCG/NCC ratios	Means of NXG/NXC ratios	*P* value
Chlorophyta	*O. viridis*	1.011	1.690	0.3865
	O. tauri	1.276	1.170	0.7715
	M. stagnorum	0.857	0.916	0.7728
	P. akinetum	0.900	1.147	0.2482
Charophyta	*E. fimbriata*	0.691	1.267	0.0833
	M. viride	0.647	1.588	0.2482
	C. globosum	1.206	1.102	0.7728
	C. vulgaris	0.708	1.117	0.5637
Bryophyte	*P. laevis*	0.785	1.114	0.0833
	M. aenigmaticus	0.832	0.924	0.5637
	T. lacunosa	0.755	1.036	0.0833
	M. polymorpha	0.713	1.086	0.0433
	P. patens	0.572	0.995	0.2482
	A. rugelii	0.624	1.004	0.0833
Pteridophyte	*H. squarrosa*	0.749	0.965	0.0209
Gymnosperms	*C. taitungensis*	0.786	1.227	0.0433
Monocotyledon	*B. umbellatus*	0.583	1.210	0.0209
	O. sativa	0.664	1.198	0.0209
	Z. mays	0.706	1.145	0.0209
	S. bicolor	0.655	1.197	0.0202
Dicotyledon	*B. vulgaris*	0.668	1.053	0.0209
	N. tabacum	0.644	1.180	0.0209
	A. thaliana	0.676	1.133	0.0209
	G. max	0.595	1.122	0.0209
