# Supplementary material for: Synonymous Codon Usage Bias in Plant Mitochondrial Genes Is Associated with Intron Number and Mirrors Species Evolution
Source: PLoS One. 2015 Jun 25;10(6):e0131508. doi: 10.1371/journal.pone.0131508 (PMC4481540; doi:10.1371/journal.pone.0131508)
Supplement: S8 Table — The absolute values greater than 0.6 were selected. (PDF) [file pone.0131508.s008.pdf]

S8 Table. The correlation coefficients of codons to principal components

	PC1	PC2	PC3
TTT	-0.9052		
AAA	-0.8847		
GTT	-0.8534		
TTA	-0.8388		
ATT	-0.7052		
AAT	-0.6961		
TAT	-0.6754		
GCA	-0.6145		
AGT	-0.6088		
CGT		-0.7897	
GGA		-0.7470	
GAA		-0.7073	
GTA		-0.6823	
ACT		-0.6581	
GGT		-0.6478	
CAT			-0.9060
CAC			0.6973
TAC			0.8166
AAC			0.8605
CGC		0.6400	
GCG		0.7043	
AGC		0.7371	
TTG		0.7216	
CAG		0.7749	
TGC		0.8348	
GGC		0.8551	
CCA	0.6090		
GAC	0.6318		
ACG	0.6437		
CTC	0.6594		
GGG	0.6718		
GCC	0.6786	0.6668	
CTG	0.6791	0.6347	
AGA	0.7200		
GTC	0.7288		
GAG	0.7605		
AGG	0.7694		
ACC	0.7838		
TTC	0.8174		

CCG	0.8329
ATC	0.8336
CGG	0.8557
CCC	0.8750
TCG	0.9012
TCC	0.9192
