# Supplementary material for: Synonymous Codon Usage Bias in Plant Mitochondrial Genes Is Associated with Intron Number and Mirrors Species Evolution
Source: PLoS One. 2015 Jun 25;10(6):e0131508. doi: 10.1371/journal.pone.0131508 (PMC4481540; doi:10.1371/journal.pone.0131508)
Supplement: S9 Table — The bias to A-ending stop codons and internal stop codons was analyzed using the numbers of TAA+TGA and TAG with the chi square (χ2) test. (PDF) [file pone.0131508.s009.pdf]

S9 Table. The statistic analysis of usage bias of stop codons and internal stop codons

Taxomony	Species	Stop codons			Internal stop codons		
		TAA+TGA	TAG	*P* value	TAA+TGA	TAG	*P* value
Chlorophyta	*O. viridis*	27	9	0.003			
	O. tauri	41	2	2.72E-09			
	M. stagnorum	37	10	8.20E-05			
	P. akinetum	51	21	4.07E-04			
Charophyta	*E. fimbriata*	29	6	1.01E-04			
	M. viride	39	2	7.54E-09			
	C. globosum	37	9	3.65E-05			
	C. vulgaris	38	8	9.72E-06			
Bryophyte	*P. laevis*	97	18	1.75E-13	72	8	8.34E-13
	M. aenigmaticus	73	19	1.80E-08	40	5	1.81E-07
	T. lacunosa	51	19	1.31E-04	1	0	-
	M. polymorpha	52	24	0.001			
	P. patens	34	8	6.02E-05			
	A. rugelii	31	11	0.002			
Pteridophyte	*H. squarrosa*	102	40	1.96E-07	3	1	0.317
Gymnosperms	*C. taitungensis*	16	11	0.335924			
Monocotyledon	*B. umbellatus*	24	5	4.18E-04			
	O. sativa	35	16	0.008			
	Z. mays	116	46	3.80E-08			
	S. bicolor	21	10	0.048			
Dicotyledon	*B. vulgaris*	101	37	5.09E-08			
	N. tabacum	107	49	3.42E-06			
	A. thaliana	97	20	1.09E-12			
	G. max	70	17	1.33E-08			
