# Supplementary figures and images for: Synonymous Codon Usage Bias in Plant Mitochondrial Genes Is Associated with Intron Number and Mirrors Species Evolution
Source: PLoS One. 2015 Jun 25;10(6):e0131508. doi: 10.1371/journal.pone.0131508 (PMC4481540; doi:10.1371/journal.pone.0131508)

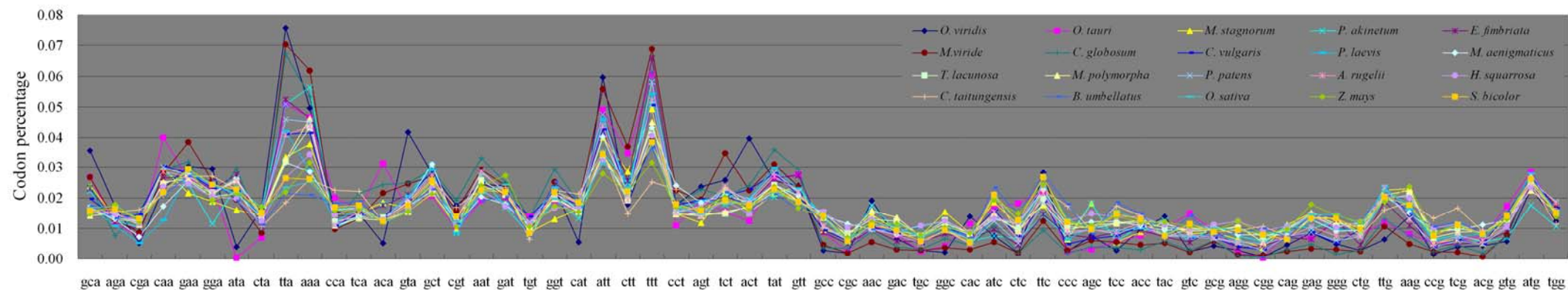

**S1 Fig.**

Supplement: S1 Fig — The index is defined as the number of each codon to the number of total 61 codons. (PDF) [file pone.0131508.s010.pdf]

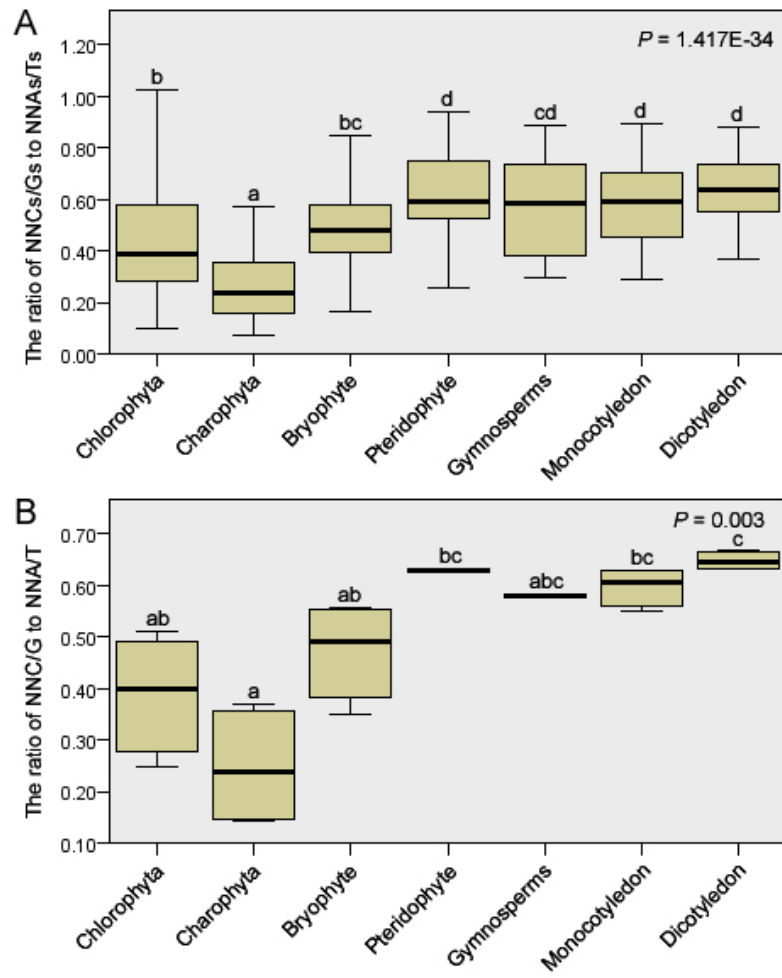

S2 Fig.

Supplement: S2 Fig — The ratios of NNCs/Gs to NNAs/Ts of 18 amino acids (A) and the ratios of NNC/G to NNA/T (B) are used for analysis with the stepwise comparison of Kruskal-Wallis test. The data are presented as the box plot of the ratios of different species. The boxes without the same lowercase letter mean significantly different from each other. (PDF) [file pone.0131508.s011.pdf]
